# Assessment of Genetic Diversity of Zoonotic *Brucella* spp. Recovered from Livestock in Egypt Using Multiple Locus VNTR Analysis

**DOI:** 10.1155/2014/353876

**Published:** 2014-01-06

**Authors:** Ahmed M. S. Menshawy, Marta Perez-Sancho, Teresa Garcia-Seco, Hosein I. Hosein, Nerea García, Irene Martinez, Ashraf E. Sayour, Joaquín Goyache, Ragab A. A. Azzam, Lucas Dominguez, Julio Alvarez

**Affiliations:** ^1^Faculty of Veterinary Medicine, Beni-Suef University, Shamlaa Street, Beni-Suef 62511, Egypt; ^2^Centro VISAVET, Universidad Complutense de Madrid, Avenida Puerta de Hierro, s/n, 28040 Madrid, Spain; ^3^Departamento de Sanidad Animal, Facultad de Veterinaria, Universidad Complutense de Madrid, Avenida Puerta de Hierro, s/n, 28040 Madrid, Spain; ^4^Department of Brucellosis, Animal Health Research Institute, Nadi-Elsaed Street, Dokki, Giza 12618, Egypt; ^5^Servicio de Microbiología, Instituto Ramón y Cajal de Investigación Sanitaria (IRYCIS), Carretera Colmenar Viejo, km. 9.100, 28034 Madrid, Spain

## Abstract

Brucellosis is endemic in most parts of Egypt, where it is caused mainly by *Brucella melitensis* biovar 3, and affects cattle and small ruminants in spite of ongoing efforts devoted to its control. Knowledge of the predominant *Brucella* species/strains circulating in a region is a prerequisite of a brucellosis control strategy. For this reason a study aiming at the evaluation of the phenotypic and genetic heterogeneity of a panel of 17 *Brucella* spp. isolates recovered from domestic ruminants (cattle, buffalo, sheep, and goat) from four governorates during a period of five years (2002–2007) was carried out using microbiological tests and molecular biology techniques (PCR, MLVA-15, and sequencing). Thirteen strains were identified as *B. melitensis* biovar 3 while all phenotypic and genetic techniques classified the remaining isolates as *B. abortus* (*n* = 2) and *B. suis* biovar 1 (*n* = 2). MLVA-15 yielded a high discriminatory power (*h* = 0.801), indicating a high genetic diversity among the *B. melitensis* strains circulating among domestic ruminants in Egypt. This is the first report of the isolation of *B. suis* from cattle in Egypt which, coupled with the finding of *B. abortus*, suggests a potential role of livestock as reservoirs of several zoonotic *Brucella* species in the region.

## 1. Introduction

Since the first description of *B. melitensis* in Malta in 1897 [1], small ruminant brucellosis (SRB) has become a widespread problem in most Mediterranean countries as well as in other parts of the world (Middle East, Central Asia, and Latin America) [2].

In spite of the lack of precise information on the prevalence of ruminant brucellosis in Egypt, the disease is considered endemic in animals and humans in most parts of the country [3] leading to an estimated yearly economic loss of approximately 60 million Egyptian pounds [4]. Several studies have attempted to determine the incidence of brucellosis in ruminants and humans in some regions of the country leading to a high variability of estimates depending mainly on the analyzed host species, geographic localization, and the serological technique used [5–8]. Predominance of smallholdings that favor close contacts between humans and animals, presence of mixed populations of animals, and consumption of unpasteurized milk and dairy products are among the main major risk factors for *Brucella* infection present in Egypt [3, 7, 9]. Implementation of control measures of bovine brucellosis (test and slaughter, S19 vaccination) since the 1980's in the country led to a reduction on *B. abortus* incidence in cattle [3]. However, efforts directed to control small ruminant brucellosis have been less intensive, contributing to an increase of *B. melitensis* infection (considered the predominant *Brucella* species in Egypt nowadays) not only in sheep and goats, but also in cattle, buffaloes, and camels [3]. The identification and molecular characterization of prevailing *Brucella* species are a cornerstone to understand the epidemiology of the disease in a region and implement adequate strategies to control this important zoonosis [10]. For this reason, a study to evaluate the heterogeneity of *Brucella *spp. isolates recovered from domestic ruminants in different governorates of Egypt was conducted.

## 2. Materials and Methods

**Figure 1:**
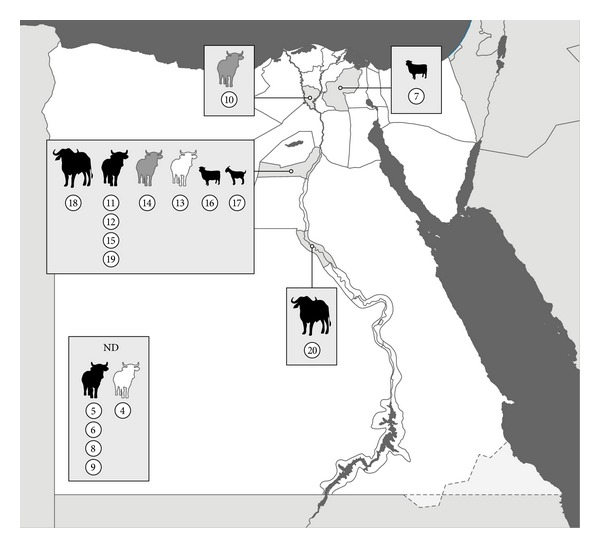
Geographical origin of the 17 *Brucella* spp. isolates (labeled from 4 to 20) recovered from livestock (buffalo, cattle, sheep, and goats) in Egypt during 2002–2007 (color of the animal indicates the *Brucella* species: *Brucella melitensis*, black*; B. suis*, grey animals, *B. abortus*, white).

In 2002–2007 a total of 17 *Brucella* isolates were cultured according to OIE guidelines from samples (milk, aborted fetus, lymph node, and spleen) of domestic ruminants (buffalo, sheep, goat, and cattle) collected by convenience sampling in Assiut, Menofia, Beni-Suef, and Sharkia Governorates (*n* = 12) and unknown locations (*n* = 5) (Table 1, Figure 1). All animals were reactors to Buffered Acidified Plate Antigen Test (BAPAT), Rose Bengal Test (RBT), Tube Agglutination Test (TAT), Rivanol Test, and Complement Fixation Test (CFT). All *Brucella*-like isolates obtained in the following 14 days were classified using phenotypical methods (morphology, CO_2_ requirements, H_2_S production, urease, catalase and oxidase activity, nitrate reduction, lactose fermentation, citrate utilization, grow in presence of thionine and fuchsin dyes (at different concentrations: 1 : 50,000 and 1 : 100,000), lysis by Tbilisi phage and agglutination with A and M anti-sera) in the Animal Health Research Institute (Giza, Egypt). *Brucella* DNA from all isolates was sent to the VISAVET Health Surveillance Centre for genetic identification and characterization. *Brucella* spp. identification was confirmed using a *Brucella*-specific PCR [11] and isolates were further characterized using the Bruce-ladder kit (Ingenasa, Tres Cantos, Spain). Isolates identified as *Brucella suis* were also analyzed using the Ingene Bruce-ladder Suis (Ingenasa) for serovar determination [12]. Finally, the whole panel was subjected to Multilocus Variable Number Tandem-Repeat analysis (MLVA-15) as described before [13]. The number of repetitions found in each locus was determined by band size assessment (according to Le Flèche et al. [13] instructions) and sequencing. Allelic diversity for each locus was determined according to Selander et al. [14] (adapted from Nei [15]). The genetic diversity was also calculated for *B. melitensis* isolates. Results were compared with those available in the database of *Brucella* from other African and Middle East countries (http://mlva.u-psud.fr/mlvav4/genotyping/view.php). All MLVA profiles not previously described have been submitted to the MLVA database (http://mlva.u-psud.fr/mlvav4/genotyping/). A cluster analysis was performed using Neighbor Joining Analysis calculating the proportion of loci at which dissimilar alleles occur using MLST Data Analysis-Tree drawing (http://pubmlst.org/cgi-bin/mlstanalyse/mlstanalyse.pl?site=pubmlst&page=treedraw&referer=pubmlst.org).

## 3. Results and Discussion

Ruminant brucellosis is an endemic food-borne disease in most parts of Egypt and other developing countries of Africa. Recent studies [10, 16, 17] have highlighted the need of identifying the animal species infected with members of the genus *Brucella* to define their potential role in the transmission of this zoonotic pathogen and to determine the prevailing *Brucella* strains present in a region in order to adopt the most suitable control strategies.

Most (13/17) of the isolates recovered from ruminants in several governorates of Egypt were identified as *B. melitensis* biovar 3 in agreement with previous reports that described this *Brucella* species as the most prevalent in Egypt [3, 18] (Table 1). However, the unexpected phenotypic results (H_2_S production, urease, grow in presence of thionine and fuchsin dyes (at different concentrations: 1 : 50,000 and 1 : 100,000), lysis by Tbilisi phage, and agglutination with A and M anti-sera) of a subset of isolates (*n* = 4) suggested their identification as non-*B. melitensis *(Table 2). Molecular identification using the Bruce-ladder kit identified in fact isolates 10–14 and 4–13 as *B. suis* and *B. abortus*, respectively. The Ingene Bruce-ladder Suis kit further identified the *B. suis* isolates as biovar 1.

*B. suis* isolates were cultured from milk (strain 10) and lymph node (strain 14) from two cows from Menofia and Beni-Suef Governorates, respectively. MLVA-15 analyses (Table 1) revealed that both isolates had typical but different *B. suis* biovar 1 patterns [13]. Zoonotic *B. suis* has been isolated in cattle elsewhere and it is becoming an emerging problem in several countries as Brazil and Colombia [19]. In this host species *B. suis* infection appears to be asymptomatic although biovar 1 shedding in milk has been described before [20] in agreement with our results. Although *B. suis* biovar 1 presence in swine has been reported previously in Egypt, its current distribution is unknown [21], and it had not been reported in cattle before. Brucellosis infection in swine has been described in the country usually by means of serological techniques [3, 22] that cannot distinguish between infections by the different *Brucella* spp. In Egypt, swine (with an approximate population of 30,000 animals [3]) may live in small groups in contact with other animals and humans [23]. As mentioned for *B. melitensis*, *B. suis *could be easily transmitted from swine to other animals and humans in these small holdings. To our knowledge, this is the first detection of the zoonotic biovar 1 of *Brucella suis* in cattle in Egypt. No information was available regarding potential contact between swine and the cattle from which *B. suis* was recovered in our study. However, taking into account the presence of a zoonotic *B. suis *biovar 1 in the region and the high number of reactors reported previously in swine populations located in different areas of Egypt (up to 12.61% using RBT [23]), more efforts are needed in order to determine the importance of this animal species as a source of infection and to avoid spillover to other domestic animals and human.

The presence of *B. abortus* in cattle in Egypt was also demonstrated here in agreement with previous occasional reports [3]. One of the two different MLVA-15 patterns (Table 1) found in the two *B. abortus* strains matched existing profiles in the MLVA Bank-Microbes genotyping [13] including *B. abortus* RB51 strains [24, 25] isolated in USA, Italy, and Portugal. However, results of the Bruce-ladder kit ruled out a possible isolation of this vaccine strain. The other MLVA profile was not present in the MLVA database.

**Figure 2:**
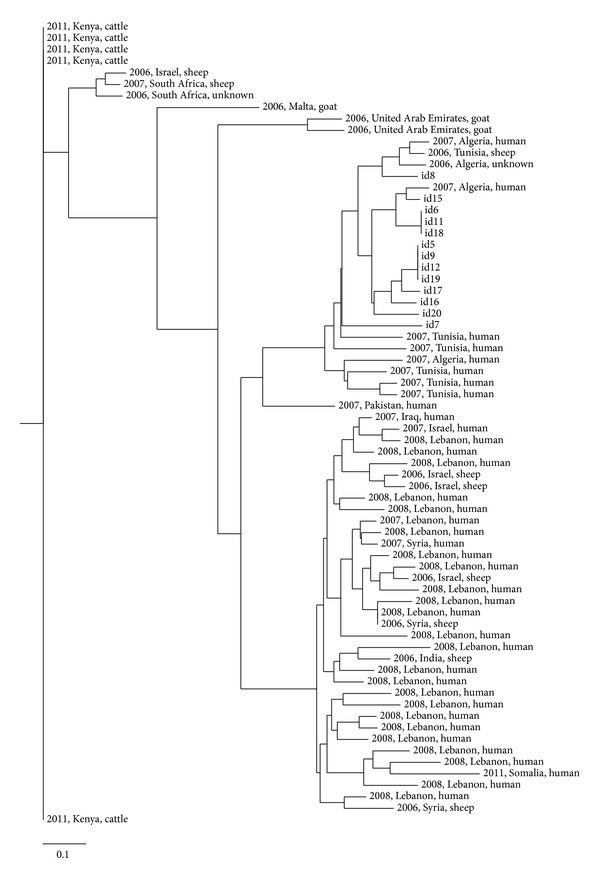
Neighbor Joining Analysis for the MLVA-15 profiles of 13 *B. melitensis* isolates recovered from domestic ruminants from Egypt in 2002–2007 compared with 57 isolates from Africa and Middle East recovered in 2006–2012 (source: http://mlva.u-psud.fr/mlvav4/genotyping/view.php).

Nevertheless, the most prevalent *Brucella* species found in the study was *B. melitensis* as previously described. All isolates belonged to West Mediterranean Group (MLVA8 genotype 51). The genetic heterogeneity existing among the 13 isolates analyzed was high (Table 1), with a total of 8 different genotypes (*h* = 0.801) (Figure 1), none of which had been included in the MLVA Bank-Microbes genotyping (*Brucella* Aggregated database, http://mlva.u-psud.fr/mlvav4/genotyping/). A high discriminatory power of MLVA-15 had been previously reported in *B. melitensis* isolates from Lebanon, Spain, and China [26–28]. In our study, the highest genetic heterogeneity was found in markers bruce 09 and bruce 16 (*h* = 0.712 and *h* = 0.596, resp.) while only one allele was found in MLVA markers bruce 21 and bruce 30 from panel 2 and in all markers from panel 1. However, the Neighbor Joining Analysis clustered the Egyptian profiles obtained in this study with *B. melitensis* isolates in a large cluster with profiles from sheep and human isolates recovered in Algeria and Tunisia (Figure 2). Some authors have previously reported the limited value of panel 1 and panel 2A [27] to differentiate *B. melitensis* isolates recovered from the same geographical origin/outbreak [27, 28]. Our results also confirm the need of using markers of panel 2B to achieve a sufficient discriminatory capacity when isolates are geographically related.

## 4. Conclusions

The high genetic heterogeneity found in this study and particularly the identification of zoonotic strains of *B. suis* and *B*. *abortus* isolated from samples from domestic ruminants suggest a complex underlying epidemiological situation in Egypt. In addition, our results demonstrate the usefulness of a complete phenotypic and genetic characterization of isolates to avoid misclassification of bacterial species belonging to the *Brucella* genus. Our study, although performed on a limited sample size, gives an insight in the current disease-causing *Brucella* species present in domestic ruminants in Egypt. Further studies aiming at the assessment of the prevalence of *B. suis* in domestic ruminants and swine in Egypt using adequate identification techniques would be needed in order to determine the importance of the infection due to this zoonotic pathogen in livestock in the region.

## Figures and Tables

**Table 1:** * Brucella* spp. isolates recovered from livestock in Egypt (2002–2007) and examined by MLVA-15 technique included in the present study (*n* = 17). Panel 1 includes eight minisatellite markers (Bruce 06–Bruce 55) and Panel 2 is composed of seven microsatellite markers (Bruce 18–Bruce 30). The number of tandem repeats observed in each marker is indicated in the corresponding cells.

Id. strain	Sample	Host	Origin	Year	Specie (biovar)	MLVA-15 Panel 1								MLVA-15 Panel 2						
						Bruce 06	Bruce 08	Bruce 11	Bruce 12	Bruce 42	Bruce 43	Bruce 45	Bruce 55	Bruce 18	Bruce 21	Bruce 04	Bruce 07	Bruce 09	Bruce 16	Bruce 30
5	Lymph node	Cow	ND*	2002–2007	*B. melitensis* bv. 3	3	5	3	13	1	1	3	3	8	8	7	5	9	5	3
6	Fetus	Cow	ND	2002–2007	*B. melitensis *bv. 3	3	5	3	13	1	1	3	3	8	8	7	5	6	4	3
7	Lymph node	Sheep	Sharkia	2007	*B. melitensis *bv. 3	3	5	3	13	1	1	3	3	7	8	5	7	5	8	3
8	Spleen	Cow	ND	2002–2007	*B. melitensis *bv. 3	3	5	3	13	1	1	3	3	7	8	6	5	8	4	3
9	Lymph node	Cow	ND	2006-2007	*B. melitensis *bv. 3	3	5	3	13	1	1	3	3	8	8	7	5	9	5	3
11	Lymph node	Cow	Beni-Suef	2006-2007	*B. melitensis *bv. 3	3	5	3	13	1	1	3	3	8	8	7	5	6	4	3
12	Lymph node	Cow	Beni-Suef	2006-2007	*B. melitensis *bv. 3	3	5	3	13	1	1	3	3	8	8	7	5	9	5	3
15	Lymph node	Cow	Beni-Suef	2006-2007	*B. melitensis* bv. 3	3	5	3	13	1	1	3	3	8	8	7	5	7	4	3
16	Lymph node	Sheep	Beni-Suef	2006-2007	*B. melitensis* bv. 3	3	5	3	13	1	1	3	3	8	8	6	5	9	5	3
17	Lymph node	Goat	Beni-Suef	2006-2007	*B. melitensis* bv. 3	3	5	3	13	1	1	3	3	8	8	7	5	10	5	3
18	Milk	Buffalo	Beni-Suef	2006-2007	*B. melitensis* bv. 3	3	5	3	13	1	1	3	3	8	8	7	5	6	4	3
19	Fetus	Cow	Beni-Suef	2006-2007	*B. melitensis* bv. 3	3	5	3	13	1	1	3	3	8	8	7	5	9	5	3
20	Milk	Buffalo	Assiut	2007	*B. melitensis *bv. 3	3	5	3	13	1	1	3	3	8	8	6	5	6	6	3

10	Milk	Cow	Menofia	2007	*B. suis* bv. 1	2	3	6	10	4	1	5	2	4	9	6	6	5	5	3
14	Lymph node	Cow	Beni-Suef	2006-2007	*B. suis* bv. 1	2	3	6	10	4	1	5	2	4	9	5	5	8	6	3

13	Spleen	Cow	Beni-Suef	2006-2007	*B. abortus *	4	5	4	12	2	3	3	3	6	8	3	7	3	3	5
4	Milk	Cow	ND	2002–2007	*B. abortus *	4	5	4	12	2	3	3	3	6	8	3	9	3	3	5

*ND: non determined.

**Table 2:** Differential phenotypic characteristics of *Brucella suis* (strains 10 and 14) and *B. abortus* (strains 4 and 13) isolated from cattle in Egypt, 2003–2007. Three reference strains (*B. melitensis* strain Ether, *B. suis* strain 1330, and *B. abortus* strain 544) are included for comparison.

	*B. melitensis *strain Ether	*B. suis* strain 1330	*B. abortus* strain 544	Strain 10/14 (*B. suis*)	Strain 4/13 (*B. abortus*)
H_2_S production	−	+++	+	+++	+++
Urease activity	+ in 18–24 h	++ in <15 min	+ in 2 h	++ in 3–5 min	+ in 2 h
Growth in presence of dye thionin 1 : 50000	+	+	−	+	−
Growth in presence of dye thionin 1 : 100000	+	+	−	+	−
Growth in presence of dye fuchsin 1 : 50000	+	−	+	−	+
Growth in presence of dye fuchsin 1 : 100000	+	−	+	−	+
Agglutination with A anti-sera	+	+	+	+	+
Agglutination with M anti-sera	+	−	−	−	−
